# Personalized Help-Seeking Web Application for Chinese-Speaking International University Students: Development and Usability Study

**DOI:** 10.2196/35659

**Published:** 2023-02-17

**Authors:** Isabella Choi, Gemma Mestroni, Caroline Hunt, Nick Glozier

**Affiliations:** 1 Central Clinical School Faculty of Medicine and Health The University of Sydney Camperdown Australia; 2 Australian Research Council Centre of Excellence for Children and Families Over the Life Course Sydney Australia; 3 School of Psychology Faculty of Science The University of Sydney Sydney Australia; 4 Brain and Mind Centre The University of Sydney Sydney Australia

**Keywords:** help seeking, mental health, international students, Chinese international students, mental health awareness

## Abstract

**Background:**

The mental health of international students is a growing concern for education providers, students, and their families. Chinese international students have low rates of help seeking owing to language, stigma, and mental health literacy barriers. Web-based help-seeking interventions may improve the rate of help seeking among Chinese international students.

**Objective:**

This study aimed to describe the development of a mental well-being web app providing personalized feedback and tailored psychoeducation and resources to support help seeking among international university students whose first language is Chinese and test the web application’s uptake and engagement.

**Methods:**

The bilingual *MindYourHead web application* contains 6 in-app assessments for various areas of mental health, and users are provided with personalized feedback on symptom severity, psychoeducation tailored to the person’s symptoms and information about relevant interventions, and tailored links to external resources and mental health services. A feasibility study was conducted within a school at the University of Sydney to examine the uptake and engagement of the web application among Chinese international students and any demographic characteristics or help-seeking attitudes or intentions that were associated with its engagement.

**Results:**

A total of 130 Chinese international students signed up on the web application. There was an uptake of 13.4% (122/908) in the schools’ Chinese student enrollment. Most participants (76/130, 58.5%) preferred to use the web application in Chinese and used informal but not formal help for their mental health. There was considerable attrition owing to a design issue, and only 46 students gained access to the full content of the web application. Of these, 67% (31/46) of participants completed 1 or more of the in-app mental well-being assessments. The most commonly engaged in-app assessments were distress (23/31, 74%), stress (17/31, 55%), and sleep (15/31, 48%), with the majority scoring within the moderate- or high-risk level of the score range. In total, 10% (9/81) of the completed in-app assessments led to clicks to external resources or services. No demographic or help-seeking intentions or attitudes were associated with web-application engagement.

**Conclusions:**

There were promising levels of demand, uptake, and engagement with the *MindYourHead* web application. The web application appears to attract students who wished to access mental health information in their native language, those who had poor mental health in the past but relied on informal support, and those who were at moderate or high risk of poor mental well-being. Further research is required to explore ways to improve uptake and engagement and to test the efficacy of the web application on Chinese international students’ mental health literacy, stigma, and help seeking.

## Introduction

### Help-Seeking Among Chinese-Speaking International Students

Psychological distress is common among university students. Approximately one-third of students have a common mental disorder during their university education, and of those, only 25% receive treatment [1]. Before the pandemic, international students accounted for approximately 25% of university enrollments in Australia, with the majority coming from China (38%), followed by India (18%) and Nepal (7%) [2]. International students in Australia have similar levels of psychological distress as domestic students [3-5], yet they are significantly less likely to access mental health services [6-8]. A survey found that 17% of international students sought help from a health professional for a mental health problem compared with 55% of domestic students at an Australian university [4]. Similarly, another web-based survey of students at an Australian university and associated tertiary college found that only 21% of international students reported having previously used a mental health service compared with 58% of domestic students [6]. International students reported more negative attitudes and lower intentions for seeking help for mental health concerns compared with domestic students [6]. As the number of international students is likely to rebound after COVID-19, it is vital to support help seeking in this population.

Low rates of help seeking for mental health problems are evident among Chinese-speaking international students, with 1 survey finding that more than half of Chinese-speaking international students in Australia reported high psychological distress, yet only 9% sought help from mental health services [7]. Common barriers to help seeking among Chinese-speaking international students included not recognizing their symptoms as part of mental health problems, thinking their problem was not severe or serious enough, and not being aware of the available treatment services [7]. In addition, Chinese migrants have high levels of stigmatizing attitudes toward mental illness [9], and fear of *losing face* can hinder their help seeking [10]. They can face practical barriers such as concerns about the cost of services or transportation difficulties, lack of time, and language barriers [7]. Although all international students attending university in Australia must possess a certain level of English proficiency, nonnative English speakers can find it difficult to understand mental health–related issues or express their concerns in a second language owing to fears of not being understood or feelings of embarrassment or shame [11,12]. As such, many Chinese-speaking international students preferred to handle the problem alone or rely on an intimate partner, family, or friends for help [13].

### Digital Help-Seeking Interventions

Help-seeking interventions aim to improve help-seeking attitudes, intentions, and behaviors through delivering information to improve mental health literacy; reducing stigma surrounding mental disorders; providing help-seeking source information about how and where to find potential providers of help; and, to a lesser extent, providing personalized feedback about the individual’s symptoms to prompt help seeking among those with higher symptom levels [14]. A meta-analysis found that help-seeking interventions improved attitudes, intentions, and behaviors to seek formal help for mental health problems, with long-term benefits for formal help-seeking behaviors [15]. A recent systematic review also provided support that digital help-seeking interventions can improve help-seeking intentions, but more research is needed on actual help-seeking behaviors [16].

There is some support for digital interventions improving help seeking among university students [17-22]. A web-based psychoeducation intervention provided university students with vignettes of a young person experiencing a mental health problem, alongside with a description of symptoms and treatment options, and challenging stigmatizing views [21]. There was an increase in mental health literacy and a decrease in mental health stigma in the intervention group compared with the control group [21]. In addition, the changes in the level of knowledge and stigma were associated with more positive help-seeking attitudes and intentions [21]. In the German site of the World Health Organization’s World Mental Health International College Student initiative, university students were randomized to either an intervention condition or a control condition. Students in the intervention condition additionally received personalized feedback based on symptom severity, psychoeducation tailored to the personal symptom profile, and information about mental health services [18]. Those who received personalized feedback on their mental health were more likely to report significantly higher intentions to use mental health support services in the next semester than those who did not [18]. Only 1 web-based help-seeking intervention specifically targeted international students [17]. The intervention provided information on depression symptoms, treatment, and help options, which improved help-seeking attitudes but not help-seeking intentions [17].

### Objectives

Although these findings are promising, it is unclear whether such effects will generalize to a Chinese-speaking international student population that faces additional language and attitudinal barriers to help seeking. It is important to consider the language, culture, and context when designing interventions for people from culturally diverse backgrounds, and there is evidence that suggests that highly culturally adapted digital mental health interventions are more efficacious [23]. This paper describes the development and feasibility study of a bilingual help-seeking digital intervention that provides personalized feedback and psychoeducation to support help seeking among Chinese-speaking international university students. The feasibility study aims to examine the uptake and engagement of the intervention among Chinese-speaking international students and to explore if any participant demographic characteristics, help-seeking attitudes, and help-seeking intentions were associated with its engagement.

## Methods

### Development Overview

We developed a minimum viable product (MVP) of a bilingual mental well-being help-seeking website application, *MindYourHead,* for Chinese first-language international students. The design of the web application was guided by the dynamic process model of help seeking by Rickwood et al [24], whereby help seeking begins with the awareness of symptoms and appraisal of having a problem, followed by the expression of symptoms and need for support; the identification of accessible help sources; and, finally, the willingness to seek help and disclose relevant information [24]. Informed by this model, the *MindYourHead* web application was designed to address help-seeking barriers relevant to Chinese international students by providing (1) personalized feedback based on individual symptom severity to address low symptom awareness, (2) psychoeducation tailored to symptoms and information about interventions appropriate to their symptom level to address the barriers of low mental health literacy and beliefs about the helpfulness of interventions, (3) tailored links to external resources and mental health services to address potential barriers of insufficient knowledge about mental health services and ease of access, (4) psychoeducation addressing common questions about mental health and help seeking and issues such as cost and confidentiality, and (5) links to crisis support services. The web application was available in simplified Chinese and English to address language barriers. The web application could be accessed by a mobile phone, tablet, and desktop.

The content for the web application was developed through a review of the relevant literature on common mental health issues faced by international students as well as consultation with the target population through user design workshops. A series of 4 user design workshops were conducted with Chinese-speaking international students. In the initial workshops, the students were presented with the objective and in-principal design of the web application. They were asked to generate areas of mental well-being they were interested in self-assessing, the type of information they wished to learn from the personalized feedback, and the type of services or resources they would find helpful. The workshop also explored the language used by Chinese international students to describe their emotional and personal problems and how to make the web application engaging for students. In the subsequent workshops, students were guided to use the prototype web application through a read-aloud format, and they were asked to comment on how useful they found the feedback and resources that were provided. They also commented on the indexes of user experience that were used to refine the layout and functionality of the web application. User feedback informed the final MVP design, but workshop findings are not presented as no ethical approval was obtained for conducting the workshops.

We also implemented many of the recommendations identified in a literature review to help guide the development of better and more rigorous apps [25]. This included providing mental health information, providing links to crisis support services, providing tailoring interventions as per the individual’s needs, using a cognitive behavioral therapy framework, recommending helpful activities outside of the app, and creating a simple and intuitive interface [25]. Another design consideration was to address core psychological needs such as autonomy, competence, and relatedness, which can improve engagement outcomes with mental health apps and could be a critical factor to consider when developing apps [26]. The *MindYourHead* web application provided autonomy by allowing users the freedom to pick and choose which content to explore in any order they choose. Feelings of competence and relatedness may be increased through the simple and intuitive design of the *MindYourHead* web application and its availability in the user’s preferred language. An international student with Chinese as their first language studying a medicine degree was involved in translating the English content developed by a clinical psychologist (IC) and a clinical psychology registrar (GM) on the team into simplified Chinese. The translations were reviewed by IC, who was also fluent in Chinese, and by Chinese-speaking international students from the workshops.

### Description of MindYourHead MVP

#### Overview

**Figure 1:**
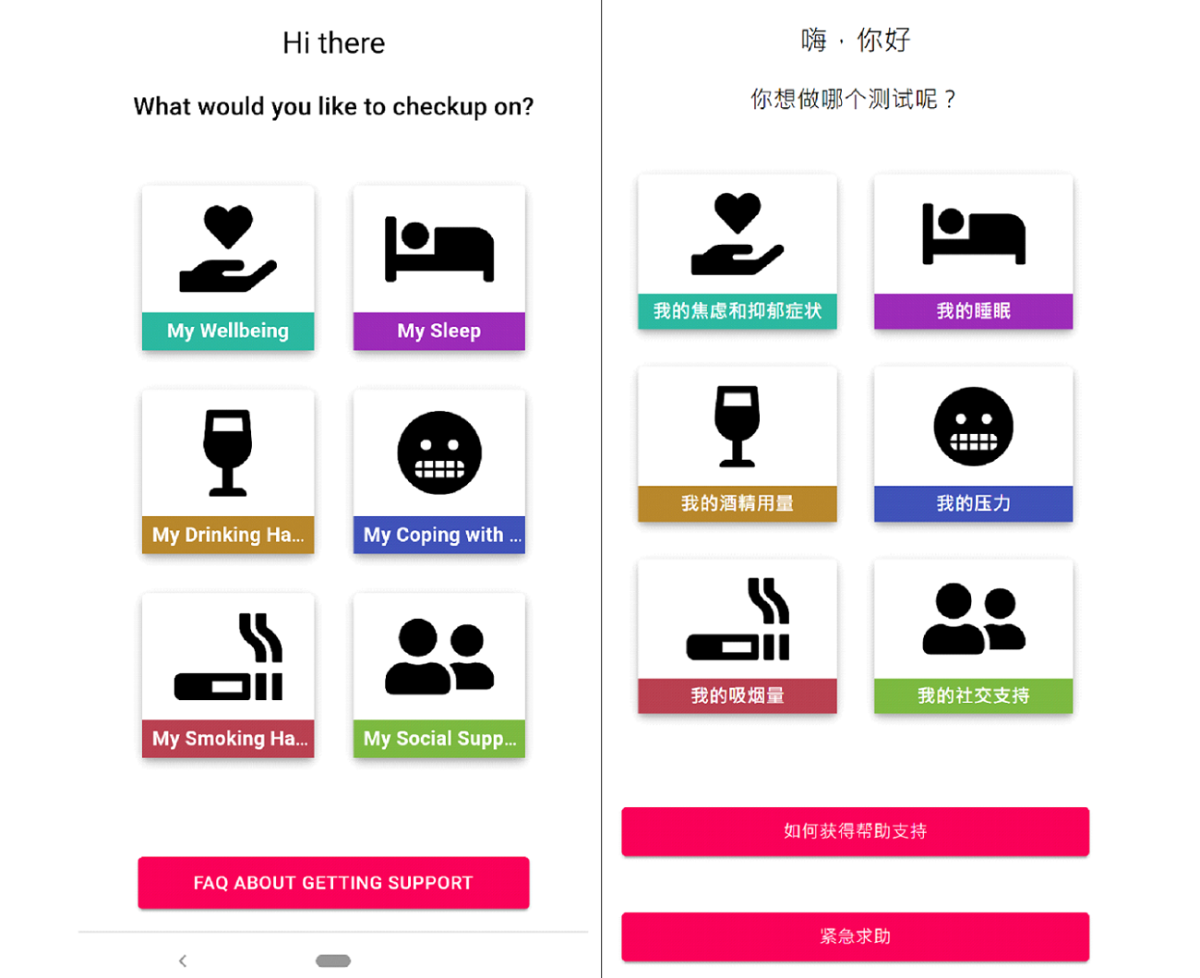
Home page (English and Chinese).

**Figure 2:**
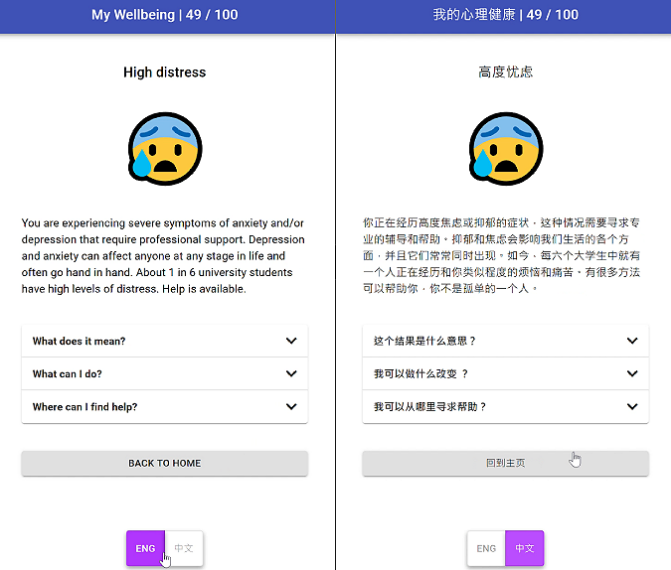
Example of feedback for high distress (English and Chinese).

The home page of *MindYourHead* presented users with a choice of 6 in-app assessments. The in-app assessments were informed by the user design workshops and used validated mental health screening questionnaires, including psychological distress, sleep, stress, alcohol consumption, smoking, and social support (Figure 1). Participants could decide which in-app assessment they wished to explore. They had the autonomy to select to complete as many or as few of the in-app assessments as they wished. On the completion of each in-app assessment, users were provided with immediate personalized feedback related to their symptom severity, brief psychoeducation tailored to symptoms and relevant interventions, and tailored links to external mental health services and web resources (Figure 2). Participants could also select whether to use the web application in English or simplified Chinese.

#### In-App Assessments

##### Distress

Distress was measured using the Kessler Psychological Distress Scale (K10) [27], a widely used and well-validated short-form measure for distress [28]. The K10 asks 10 questions related to how depressed and anxious the individual has been feeling over the last month, with scores ranging from 10 to 50. Participants were asked to indicate how often they have felt a particular way (eg, hopeless) on a scale from *1=all of the time to 5=none of the time*. The Chinese version of the K10 also demonstrated good reliability and validity [29]. The K10 was labeled as the *My Wellbeing* assessment, with a score between 10 and 15 indicating *low distress*, 16 and 29 indicating *moderate distress*, and 30 and 50 indicating *high distress*, using cutoffs from Australian K10 scoring norms [30].

##### Sleep Problems

Sleep problems were measured using the Insomnia Severity Index (ISI) [31]. The ISI asks 7 questions related to sleep problems over the past 2 weeks, with scores ranging from 0 to 28. Participants were asked to rate the severity of the problems on a scale from *0=none to 4=very severe*. The Chinese version of this scale has also been found to be reliable and valid [32]. This was labeled as the *My Sleep* assessment in the app, with scores between 0 and 14 indicating *Low Sleep Problems*, 15 and 21 indicating *Moderate Sleep Problems*, and 22 and 28 indicating *High Sleep Problems* [31].

##### Stress

Stress was labeled as *My Stress* assessment in the app. It was measured using the Perceived Stress Scale (PSS) [33]. Participants were asked to rate their thoughts and feelings over the past month across 10 items, such as “In the last month, how often have you been upset because of something that happened unexpectedly?” Each item was rated on a scale from *0=never to 4=very often*, producing a total score ranging from 0 to 40. The simplified Chinese version of this scale also has satisfactory psychometric properties [34]. A score between 0 and 13 indicated *Low Stress*, 14 and 26 indicated *Moderate Stress*, and 27 and 40 indicated *High Stress*.

##### Alcohol Consumption

Alcohol consumption was measured using the Alcohol Use Disorders Identification Test–Concise (AUDIT-C) [35], a 3-item screening measure that has been used extensively to identify those at risk for an alcohol use disorder. This short-form scale has demonstrated construct validity and internal consistency in a student population, with a Cronbach α of .80 [36]. Each item has a rating between 0 and 4 points for questions relating to how often and how much alcohol the individual consumes, and total scores range from 0 to 12. The AUDIT-C is the only alcohol screening test specifically designed for international use, and the Chinese version has been demonstrated to have satisfactory psychometric properties [37]. This was labeled as *My Drinking Habits*, with a score between 0 and 6 indicating *low-risk consumption* and 7 and 12 indicating *high-risk consumption* [36].

##### Smoking

Smoking was measured using 3 screening questions to assess nicotine dependence taken from the Fagerstrom Test for Nicotine Dependence [38]. The questions included the following: “How soon after waking do you smoke your first cigarette?” (if within 30 minutes, indicative of nicotine dependence); “How many cigarettes do you smoke on a typical day?” (if >10 a day, indicative of nicotine dependence); and “If you have previously attempted to quit, did you experience withdrawals or cravings?” A positive response to any question indicated *high risk*. The simplified Chinese version has been shown to have satisfactory reliability and validity [39]. This formed the *My Smoking Habits* assessment.

##### Social Support

Social support was labeled as *My Social Support* assessment in the app. It was measured using the Multidimensional Scale of Perceived Social Support [40]. The scale includes 12 items that ask questions related to whether the individual feels that they have strong social support from family, friends, or a significant other. Responses were made on a 7-point Likert scale from *1=very strongly disagree to 7=very strongly agree*, with total scores ranging from 12 to 84. This scale has demonstrated good reliability and validity in both the original version [41] and the Chinese version [42]. A total score of 12-35 indicated *Low Social Support*, 36-60 indicated *Moderate Social Support*, and 61-84 indicated *High Social Support*.

#### Personalized Feedback

Users received personalized risk feedback immediately after completing the questionnaires based on their score level, along with 3 levels of information: “what does this mean?” “what can I do?” and “where can I find help?” (Figure 2). Each level of feedback was tailored to the individual’s score for the relevant scale for the particular in-app assessment (eg, K10). Participants’ scores fell into one of the 2 or 3 levels (eg, high distress, moderate distress, or low distress). The feedback included information about symptoms, frequency in the student population, strategies to reduce symptoms and increase well-being, and the available evidence-based treatments. Users were provided with information and links for up to 4 relevant external mental health services or evidence-based resources (eg, university counseling service, university health service, MyCompass app [43], and ThisWayUp web-based clinic [44]). Whenever a user scored within the *high-risk* range on any measure, they were encouraged to seek support from the university counseling service and a general practitioner.

#### Other App Sections

The web application included an emergency help section that provided the telephone number and links to emergency and other 24-hour support services and translation services. The “Frequently Asked Questions About Getting Support” section addressed common questions about mental health, access to treatment, costs, and confidentiality, as guided by the user design workshops. Users could explore these sections freely.

### Feasibility Study

#### Aims

We assessed the feasibility of the *MindYourHead* web application by evaluating its uptake and engagement among Chinese-speaking international students within a school at the University of Sydney. We further aimed to explore whether any participant’s demographic characteristics and preexisting help-seeking attitudes and intentions were associated with engagement with the web application.

#### Ethics Approval

The feasibility study was approved by the Human Research Ethics Committee of the University of Sydney (project number: 2020/610).

#### Recruitment

**Figure 3:**
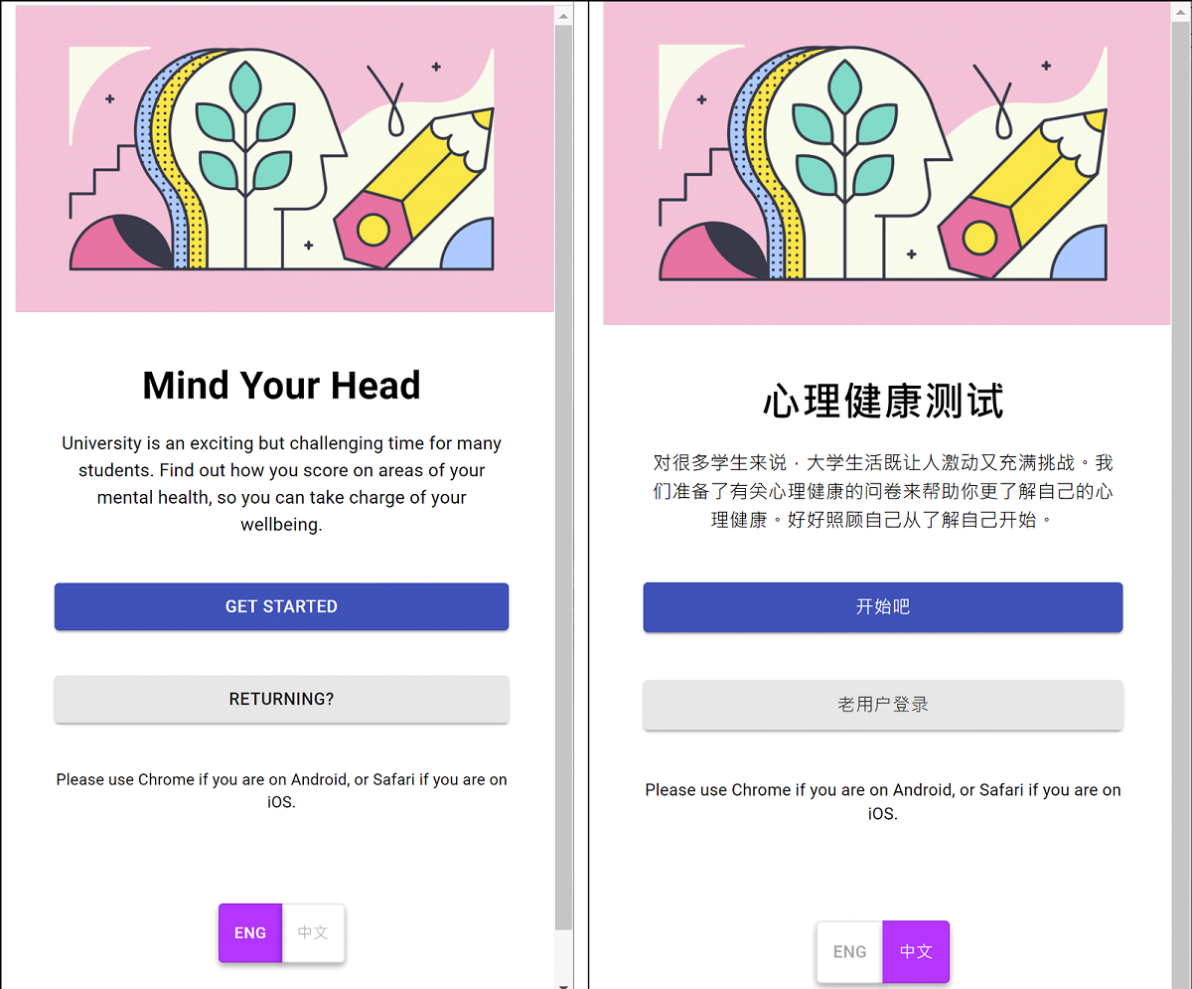
Landing page (English and Chinese).

Participants had to be Chinese-speaking international students, enrolled in a course at the University of Sydney, with access to a reliable internet connection. The recruitment was conducted through the University of Sydney’s School of Architecture, Design, and Planning to determine the uptake of the *MindYourHead* web application by a pool of potential students. The school had 908 Chinese international students (from China, Hong Kong, Macau, and Taiwan) during the semester in which this study was conducted. The school sent 3 recruitment emails to enrolled students between October 2020 and December 2020, inviting Chinese-speaking international students to participate in a study to test a mental well-being web application for Chinese international students. The email provided the students with a link to the landing page of the web application (Figure 3). No reimbursement was offered to students for participating in the study.

#### Measures

##### Baseline Self-report Questionnaires

Participants completed the demographic information before gaining access to the full content of the web application. Questions included their age; gender; home country; current location; and faculty, level, and year of study. Participants were asked to rate their English language proficiency (very well, well, not well, or not at all). Participants were also asked whether they had ever experienced a period of poor mental health in the past and were asked to indicate whether they had ever sought help for their mental health across several different sources (ie, mental health professionals, friends, web-based resources, etc).

The Attitudes Toward Seeking Professional Psychological Help Scale–Short Form (ATSPPH-SF) [45] was used as a measure of participants’ attitudes toward seeking professional help for their mental health. The scale has demonstrated internal consistency ranging from 0.82 to 0.84 among samples of college students [45]. The scale has the following 2 factors: openness to seeking treatment for emotional problems and perceived value and need in seeking treatment. Scores range from 0 to 30, with higher scores indicating a more positive treatment attitude and shown to be associated with less treatment-related stigma and greater intentions to seek treatment in the future [46]. The Chinese version of the questionnaire has demonstrated strong reliability and validity [47].

The General Help-Seeking Questionnaire (GHSQ) [48] was used to assess the intention to seek help for personal or emotional problems from different sources. The scale has demonstrated reliability and validity as a flexible measure that can be applied to various contexts (Cronbach α=.70; test-retest reliability=0.86) in both English [48] and Chinese [49]. Additional sources of help seeking relevant to the university population were included in addition to common sources of help, such as mental health websites (eg, Beyond Blue and Headspace), e–mental health apps (eg, Smiling Mind app and My Compass app), university counseling and psychology services, and university staff. Participants answered how likely they were to use various mental health services if they had a personal or emotional problem on a 7-point Likert scale from *1=extremely unlikely to 7=extremely likely*.

##### Engagement and Help-Seeking Measures

The web application automatically recorded objective engagement data, including the number of assessments completed, assessment scores and *symptom/risk level*, and link clicks to external resources.

#### Procedure

**Figure 4:**
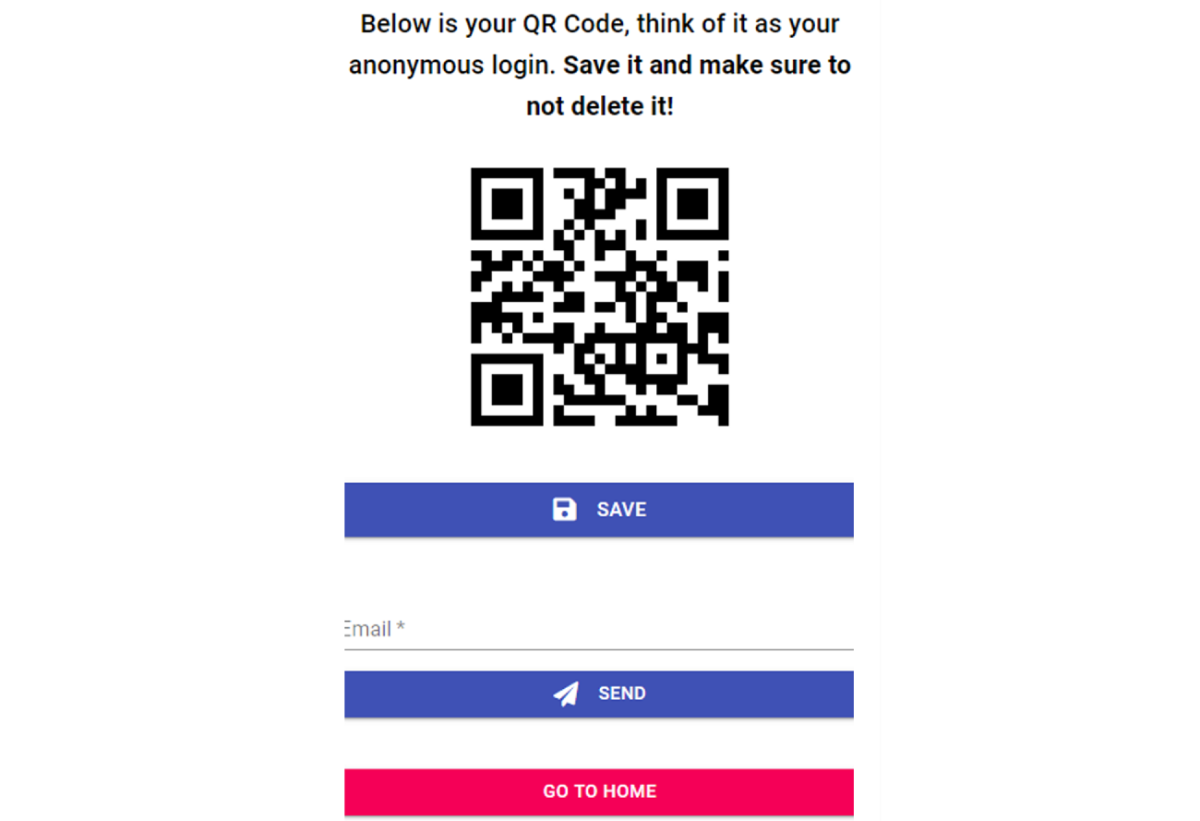
Example of the QR code page after completion of demographic questionnaires.

Participants read the participant information statement and provided informed consent within the web application. Then, they completed a demographic questionnaire, which generated a unique QR code that participants could save on their devices (Figure 4). The QR code was used as a deidentified identifier to track the participant’s data and allowed the participant to log in to their account upon return. On receiving their unique QR codes, the participants completed the baseline measures (ATSPPH-SF and GHSQ). They were then given access to the home page of the *MindYourHead* web application. This gave them access to the in-app assessments and personalized feedback. The in-app assessments were for distress (K10), sleep (ISI), stress (PSS), alcohol use (AUDIT-C), smoking (Fagerstrom Test for Nicotine Dependence), and social support (Multidimensional Scale of Perceived Social Support; Figure 1). The participants were able to freely access the content of the web application as many times as they wished and complete as many or as few of the assessments as they wished over the 3-month study period.

#### Statistical Analysis

Quantitative data were analyzed using SPSS (version 23.0; IBM). Descriptive statistics were used for participant characteristics and app use data to demonstrate the uptake and engagement of the web application. Independent 2-tailed *t* tests and Pearson chi-square tests of independence were conducted to determine whether there were any significant demographic or help-seeking intention and attitude differences between participants who gained access to the full content of the web application and those who did not and between participants with full access to the web application who engaged with at least one assessment and those who did not engage in any. Pearson correlation coefficient tests were conducted to determine whether there were any correlations between the participant’s intentions and attitudes toward help seeking (ie, scores on the ATSPPH-SF and GHSQ) and their engagement within the web application (ie, the number of in-app assessments completed). The α level was set at .05 to determine statistical significance.

## Results

### Uptake

**Figure 5:**
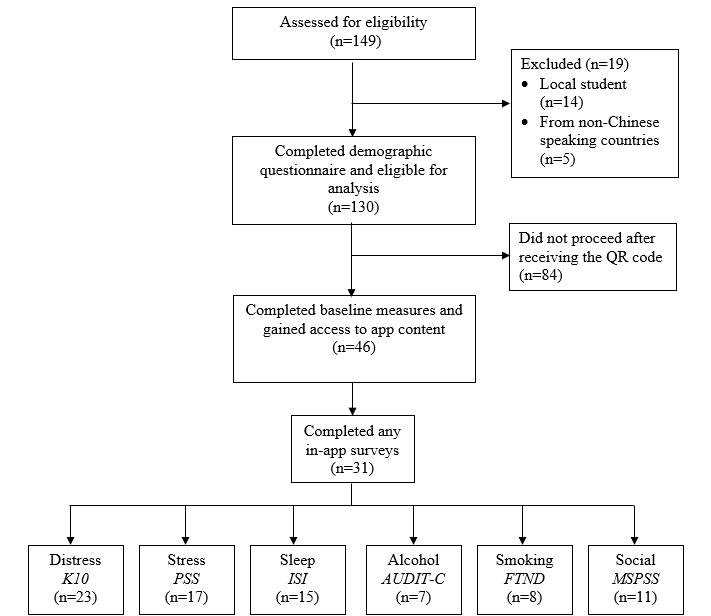
Flowchart of participant engagement at each step within the study. AUDIT-C: Alcohol Use Disorders Identification Test–Concise; FTND: Fagerstrom Test for Nicotine Dependence; ISI: Insomnia Severity Index; K10: Kessler Psychological Distress Scale; MSPSS: Multidimensional Scale of Perceived Social Support; PSS: Perceived Stress Scale.

A total of 149 participants consented to participate in the feasibility study. In total, 12.8% (19/149) did not meet the inclusion criteria and were excluded from the analysis, resulting in 130 eligible participants who met the inclusion criteria and completed the demographic questionnaire. Of the 130 participants, 122 (93.8%) were from the School of Architecture, Planning and Design, representing an uptake of 13.4% (122/908) of the school’s enrollment of Chinese international students. Figure 5 illustrates the participant flow.

### Demographic Characteristics

Table 1 presents the demographic characteristics of the participants. The mean age of the participants was 22 (SD 2.23; range 18-28) years. Most participants were women (88/130, 67.7%), from China (120/130, 92.3%), currently residing in Australia (81/130, 62.3%), studying at an undergraduate level (74/130, 56.9%), and studying full time (127/130, 97.7%). All participants indicated that they had some level of proficiency in speaking English, but 33.8% (44/130) of participants reported that they spoke English *not well*. More than half of the participants (76/130, 58.5%) chose to use the web application in Chinese. Approximately two-thirds of participants (81/130, 62.3%) indicated that they had experienced poor mental health in the past. When asked about help seeking for their mental health in the past, most participants (78/130, 60%) mentioned that they had sought help from a friend. Less than a quarter of the participants reported seeking help from a mental health professional (27/130, 20.8%) or a general practitioner (19/130, 14.6%). Few participants (6/130, 4.6%) had ever sought help for mental health issues through web-based information via websites or using e–mental health programs or apps.

**Table 1:** Demographic characteristics and significance statistics of the participants who joined the study, those with full access, and those who completed an in-app assessment (N=130).

		Total uptake (N=130)	Full access to app content (n=46)	Completed ≥1 in-app assessments (n=31)	Full access vs not gained access (n=46 vs n=84)	*P* value	Completed ≥1 in-app assessments vs none (n=31 vs n=15)	*P* value
Age (years), mean (SD)		22 (2.23)	22.3 (2.25)	22.65 (2.36)	*t*_128_=1.151	.25	*t*_44_=1.497;	.14
**Gender, n (%)**								
	Woman	88 (67.7)	32 (69.6)	23 (74.2)	*χ*^2^_1_=0.1	.84	*χ*^2^_1_=1.4	.30
	Man	39 (30)	13 (28.3)	7 (22.6)	—^a^	—	—	—
	Nonbinary	3 (2.3)	1 (2.1)	1 (3.2)	—	—	—	—
**The language used in the web application, n (%)**								
	English	54 (41.5)	8 (17.4)	4 (12.9)	*χ*^2^_1_=17.1	<.001	*χ*^2^_1_=1.3	.41
	Chinese	76 (58.5)	38 (82.6)	27 (87.1)	—	—	—	—
**Home country, n (%)**								
	China	120 (92.3)	44 (95.6)	30 (96.8)	N/A^b^	N/A	N/A	N/A
	Hong Kong	7 (5.4)	1 (2.2)	0 (0)	—	—	—	—
	Other (Taiwan or Singapore)	3 (2.3)	1 (2.2)	1 (3.2)	—	—	—	—
**Currently in Australia, n (%)**								
	Yes	81 (62.3)	28 (60.9)	19 (61.3)	*χ*^2^_1_=0.1	.85	*χ*^2^_1_=0.0	>.99
	No	49 (37.7)	18 (39.1)	12 (38.7)	—	—	—	—
**Number of years in Australia, n (%)**								
	<1	34 (26.2)	11 (23.9)	8 (25.8)	*χ*^2^_3_=1.1	.78	*χ*^2^_3_=5.9	.12
	1-2	34 (26.2)	14 (30.4)	7 (22.6)	—	—	—	—
	2-4	30 (23.1)	9 (19.6)	5 (16.1)	—	—	—	—
	>4	32 (24.6)	12 (26.1)	11 (35.5)	—	—	—	—
**Currently studying, n (%)**								
	Undergraduate	74 (56.9)	26 (56.5)	15 (48.4)	*χ*^2^_1_=0.0	>.99	*χ*^2^_1_=2.6	.13
	Postgraduate	56 (43.1)	20 (43.5)	16 (51.6)	—	—	—	—
**Year of study, n (%)**								
	First year	64 (49.2)	20 (43.5)	16 (51.6)	*χ*^2^_2_=1.0	.61	*χ*^2^_2_=2.6	.27
	Second year	44 (33.8)	17 (37)	10 (32.3)	—	—	—	—
	Third year or above	22 (16.9)	9 (19.6)	5 (16.1)	—	—	—	—
**Study mode, n (%)**								
	Full time	127 (97.7)	46 (100)	31 (100)	N/A	N/A	N/A	N/A
	Part time	3 (2.3)	0 (0)	0 (0)	—	—	—	—
**English proficiency, n (%)**								
	Very well	16 (12.3)	5 (10.9)	2 (6.5)	*χ*^2^_2_=0.7	.71	*χ*^2^_2_=2.3	.31
	Well	70 (53.8)	27 (58.7)	20 (64.5)	—	—	—	—
	Not well	44 (33.8)	14 (30.4)	9 (29)	—	—	—	—
**Poor mental health in the past, n (%)**								
	Yes	81 (62.3)	33 (71.7)	23 (74.2)	*χ*^2^_1_=2.7	.13	*χ*^2^_1_=0.3	.73
	No	49 (37.7)	13 (28.3)	8 (25.8)	—	—	—	—
**Previously sought help from, n (%)**								
	Mental health professional	27 (20.8)	12 (26.1)	10 (32.3)	*χ*^2^_1_=1.2	.37	*χ*^2^_1_=1.9;	.29
	General practitioner	19 (14.6)	5 (10.9)	4 (12.9)	*χ*^2^_1_=0.8	.44	*χ*^2^_1_=0.4	>.99
	Intimate partner	30 (23.1)	14 (30.4)	8 (25.8)	*χ*^2^_1_=2.2	.19	*χ*^2^_1_=1.0	.50
	Friend	78 (60)	29 (63)	23 (74.2)	*χ*^2^_1_=0.3	.71	*χ*^2^_1_=5.1	.048
	Family	29 (22.3)	12 (26.1)	8 (25.8)	*χ*^2^_1_=0.1	.82	*χ*^2^_1_=0.0	>.99
	Helpline	5 (3.8)	2 (4.3)	2 (6.5)	N/A	N/A	N/A	N/A
	Web-based resources or app	6 (4.6)	3 (6.5)	3 (9.7)	N/A	N/A	N/A	N/A
	Other	2 (1.5)	1 (2.2)	1 (3.2)	N/A	N/A	N/A	N/A

^a^Not available.

^b^N/A: not applicable.

### Engagement

#### Overall App

There was a significant dropout of participants upon receiving the QR code, with only 35.4% (46/130) of participants proceeding to complete the baseline questionnaires (ATSPPH-SF and GHSQ). Participants who selected to use the web application in Chinese were more likely to complete the baseline measures and gain access to the full content of the app than those who selected to use the app in English (*P*<.001). There were no other significant differences between participants who gained access to the full content of the app and those who dropped out (Table 1). On investigation, it appeared that many participants thought they should scan the QR code instead of clicking on the *go to home* button (Figure 4), which may have accounted for the high level of dropout.

#### In-App Assessment Completion Rates and Clicks to Resources

Of the 46 participants who gained access to the full content of the *MindYourHead* web application, 31 (67%) completed ≥1 in-app assessments (Figure 5). Among the 31 participants who engaged in any in-app assessment, 11 (24%) used 1 assessment, 4 (9%) used 2 assessments, 7 (15%) used 3 assessments, 6 (13%) used 4 assessments, 1 (2%) used 5 assessments, and 2 (4%) used all 6 assessments.

**Figure 6:**
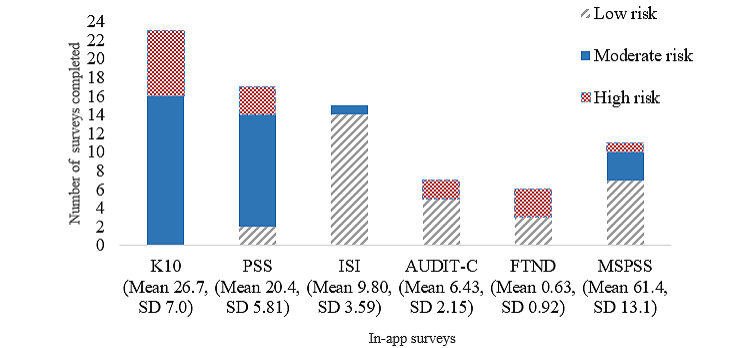
Number of in-app surveys completed for each area of content, with participant score severity shown (ie, low, moderate, or high). AUDIT-C: Alcohol Use Disorders Identification Test–Concise; FTND: Fagerstrom Test for Nicotine Dependence; ISI: Insomnia Severity Index; K10: Kessler Psychological Distress Scale; MSPSS: Multidimensional Scale of Perceived Social Support; PSS: Perceived Stress Scale.

Figure 6 shows the completion rates and participant score severity for the in-app assessments. *My Well-being* had the highest level of engagement, with 74% (23/31) of the participants completing the K10 questionnaire and 2 participants clicking on links to external resources (1 scoring moderate risk clicked on the MyCompass app [43] and 1 scoring high risk accessed the university counseling service website). This was followed by *My Stress*, with 55% (17/31) completing the PSS questionnaire and 4 participants with moderate-risk scores clicking on links to external resources (1 accessed the university counseling service, 2 accessed the This Way Up web-based clinic [44], and 1 used the Happify app [50]). *My Sleep* was used by 48% (15/31) of the participants, with no clicks to external resources. *My Social Support* was used by 35% (11/31) of the participants, and 1 individual with a low-risk score clicked on a link to the university’s Chinese Student Association. *My Smoking* was used by 26% (8/31) of the participants, and 1 high-risk scorer clicked on the link to the My QuitBuddy app [51]. *My Alcohol* was used by 23% (7/31) of the participants, with no clicks to external resources.

Of the total 81 assessments completed, there was a total of 9 (11%) link clicks to external resources. Although the moderate- and high-risk categories appeared to attract more clicks than the low-risk categories, this was not significant (*χ*^2^_2_=3.1; *P*=.22; Table 2).

**Table 2:** Total clicks by risk level across all in-app assessments (n=81).

	Total clicks, n (%)	Did not click, n (%)
High risk	2 (19)	14 (81)
Moderate risk	5 (16)	27 (84)
Low risk	1 (3)	32 (97)

#### Associations With App Engagement

There was a trend for participants to have engaged in an in-app assessment if they had previously sought help for their mental health from a friend, but this did not reach statistical significance (*χ*^2^_1_=3.7; *P*=.054). There were no other significant differences between participants who engaged with the in-app assessment content and those who did not in terms of participant characteristics and help-seeking intentions or attitudes (Table 1). There was no significant correlation between the participants’ attitudes toward seeking help for mental health and their level of engagement (Table 3).

**Table 3:** Correlation between help-seeking attitudes and intentions scores and the number of in-app assessments completed (n=31).

Variables			Values, mean (SD)		Number of assessments completed		
					Correlation coefficient, *r*		*P* value
**Intention to seek help from...**							
	Friend	5.39 (1.45)		0.04		.83	
	Partner	4.84 (1.97)		0.09		.64	
	Parent	3.58 (1.63)		0.12		.52	
	Other relative	2.55 (1.69)		0.02		.91	
	Mental health professional	4.65 (1.17)		0.27		.14	
	Physician	3.97 (1.33)		0.08		.69	
	University staff	2.74 (1.26)		0.10		.59	
	Religious leader	2.13 (1.67)		−0.34		.06	
	Helpline	2.23 (1.38)		0.06		.76	
	Web-based resources	3.48 (1.63)		0.18		.32	
	Apps	3.23 (1.33)		0.22		.23	
	No one	3.26 (1.63)		−0.30		.10	
ATSPPH-SF^a^ score			16.8 (2.17)		−0.14		.44

^a^ATSPPH-SF: Attitudes Toward Seeking Professional Psychological Help Scale–Short Form.

## Discussion

### Principal Findings

This feasibility study described the *MindYourHead* web application and examined its uptake and engagement among Chinese-speaking international students and any demographic and help-seeking characteristics associated with its engagement. The *MindYourHead* web application is a bilingual web-based help-seeking intervention that contains in-app assessments for various areas of mental health. Within each in-app assessment, users are provided with personalized feedback on symptom severity, tailored psychoeducation on symptoms and relevant interventions, and tailored links to external resources and mental health services. The uptake of the web application among Chinese-speaking international students within the school was 13.4% (122/908). The majority of participants (76/130, 58.5%) who signed up on the web application and accessed its content did so in Chinese. In total, 67% (31/46) of the participants who accessed the full app content used at least one in-app assessment, with the *distress*, *stress*, and sleep in-app assessments being the most popular. There were no demographic or help-seeking intentions or attitudes associated with engagement with the web application.

### Uptake

There is a general lack of reporting on the uptake rates of digital mental health interventions [52,53]. The uptake of digital interventions for the treatment of mental health conditions such as stress and depressive symptoms among university populations was reported to mostly range from 1% to 8% [54-59], with one study reporting 16.9% [60]. In our study, 13.4% (122/908) of the Chinese-speaking international students enrolled at the school signed up to use the *MindYourHead* web application through a passive recruitment strategy of 3 student email communications over the semester. This uptake is comparable with other personalized feedback interventions for university students, which are aimed at encouraging help seeking. A study that offered personalized assessments for depression and suicide risk reported an 8% uptake [55], and another study targeting suicide risk reported that 25% of invited students began the screening survey [20]. The World Mental Health International College Student initiative sent up to 6 reminder emails to college students, which resulted in 25.9% starting the screening survey and 12.3% completing the surveys and being eligible to receive feedback on their symptom level [18]. This feasibility study demonstrated a promising initial uptake of the *MindYourHead* web application among Chinese-speaking international students to warrant further experimental testing, although more research is needed to explore ways to improve uptake among this population and students in general.

### Engagement

Although the *MindYourHead* web application was not designed to be used in a linear fashion, and it allowed users to pick and choose the in-app assessments that were relevant or of interest to them, 67% (31/46) of the participants engaged with at least one in-app assessment in the *MindYourHead* web application, and of those, most participants (20/31, 65%) completed >1 in-app assessment. This level of participant engagement with the web application content was comparable with other digital mental health interventions, despite it not offering any symptom intervention. Approximately 21%-88% of users engaged minimally (completing at least one module or assessment) in digital self-help interventions for depression and anxiety [52]. Specifically, studies on digital mental health treatment interventions for university students have reported that 11% of participants started a web-based gratitude intervention [61], 68% opened at least one web session of a web-based therapist-assisted–acceptance-based behavioral intervention targeting anxiety [62], 74% used at least some of the unguided web-based self-help interventions (MoodGYM) [63], 76% began a web-based stress management intervention [64], and 85% completed lesson 1 of a web-based acceptance and commitment therapy prevention program [65].

Participants engaged most with content related to psychological distress, stress, and sleep but less with content related to alcohol consumption and smoking behavior. Most of the participants (48/81, 59%) who completed assessments scored in either moderate or high ranges, especially on distress and stress, suggesting that the web application attracted those who required support for early intervention or treatment. There was also a trend for those who clicked links to external resources to have scored at a moderate to a high level of risk in the in-app assessments, suggesting that the web application can support those who need to seek external support. However, the overall proportion of clicks was still low, and further qualitative research is needed to explore the reasons users click or do not click on external resources.

### Demand

This feasibility study suggests that there is a demand within this target population for a web application to support help seeking. Consistent with previous research, participants demonstrated low formal help-seeking intentions and behaviors. Despite almost two-thirds of the participants (81/130, 62.3%) having experienced poor mental health in the past, only 21% (27/130) had previously sought help from a mental health professional. On average, the participants had negative attitudes toward seeking professional help for emotional problems. Instead, the participants most commonly sought help from a friend for their mental health. Reliance on friends and self and questioning the severity of their issues were barriers to seeking professional help among Chinese international students [7]. We found that there was a trend for help seeking in the past from a friend to be associated with engagement in the in-app assessments, suggesting that the *MindYourHead* web application may support people who traditionally relied on informal help seeking by potentially linking them with formal services and resources.

This study also demonstrates the importance of developing mental health apps and resources for international students in their first language. Although international students in Australia, especially at the tertiary level, need to meet English language requirements to gain entry into their course and receive a student visa, one-third of the Chinese first-language international student participants (44/130, 33.8%) indicated that they spoke English not well. Furthermore, there was a strong preference for using the web application in Chinese among users. This is in line with past studies that have shown language barriers to be a significant obstacle in seeking help for mental health [7,66]. The level of uptake and engagement with *MindYourHead* may be owing to the web application offering mental health information in the students’ preferred language, although the low number of link clicks to external resources could be owing to the lack of multilingual resources available.

### Limitations

A major limitation was the design issue related to the QR code, which resulted in high attrition of participants before completing the baseline questionnaires and accessing the full web application. It is not known whether the help-seeking attitudes or intentions of participants who dropped out were different from those who accessed the full app content. It is possible that without confusion regarding QR codes, the actual engagement rate of the *MindYourHead* web application may have been higher. However, the dropout could also reflect that participants found the baseline questionnaires onerous to complete or that they did not know that there was more content to the app. Future versions of the *MindYourHead* app will move away from using QR codes and provide an *onboarding* of the app such that the users know what to expect. More extensive testing of prototypes with user feedback should also be conducted before commencing any future studies.

The *MindYourHead* web application was limited by the lack of multilingual resources available in Australia. Although care was taken to include Chinese-language mental health resources wherever possible, most links to external resources were only available in English, which may have limited the clicks to external resources from participants. However, this limitation likely reflects a more systemic issue regarding the paucity of multilingual mental health resources in Australia. Future versions of the app should aim to include more multilingual resources where possible or develop in-app self-help resources available in the target language.

Finally, this feasibility study focused on uptake and engagement with the app. No satisfaction or usability metrics were used, and there were no postintervention measures assessing changes in participants’ help-seeking attitudes or intentions. Furthermore, the study only measured clicks to external resources, but it is unknown whether participants actually engaged with those external resources. A randomized controlled trial is needed in the future to test the efficacy of apps in improving mental health literacy, stigma, and help-seeking intentions and attitudes as well as to qualitatively explore user experience, satisfaction, and acceptability.

### Conclusions

To the best of our knowledge, *MindYourHead* is the first mental well-being web application targeted at Chinese-speaking international students to support their help seeking and is available in both English and Chinese. This feasibility study of the *MindYourHead* web application MVP showed promising uptake and engagement among Chinese-speaking international students, comparable with other digital mental health interventions for university students. Furthermore, the web application appears to attract international students who traditionally relied on friends for mental health support by engaging them with in-app assessments and potentially directing them to formal services and resources. This study also provided useful information that can guide future versions of the web application to support international students, especially the importance of making mental health information and resources available in the target population’s native language. Further research is required to explore ways to improve uptake and engagement and to test the efficacy of the web application on Chinese international students’ help seeking in a trial.

## Abbreviations

ATSPPH-SF: Attitudes Toward Seeking Professional Psychological Help Scale–Short Form
AUDIT-C: Alcohol Use Disorders Identification Test–Concise
GHSQ: General Help-Seeking Questionnaire
ISI: Insomnia Severity Index
K10: Kessler Psychological Distress Scale
MVP: minimum viable product
PSS: Perceived Stress Scale

## References

[1] Bruffaerts R, Mortier P, Auerbach RP, Alonso J, Hermosillo De la Torre AE, Cuijpers P, Demyttenaere K, Ebert DD, Green JG, Hasking P, Stein DJ, Ennis E, Nock MK, Pinder-Amaker S, Sampson NA, Vilagut G, Zaslavsky AM, Kessler RC, WHO WMH-ICS Collaborators (2019). Lifetime and 12-month treatment for mental disorders and suicidal thoughts and behaviors among first year college students. Int J Methods Psychiatr Res.

[2] Ferguson H, Sherrel H Overseas students in Australian higher education: a quick guide. Parliament of Australia.

[3] Said D, Kypri K, Bowman J (2013). Risk factors for mental disorder among university students in Australia: findings from a web-based cross-sectional survey. Soc Psychiatry Psychiatr Epidemiol.

[4] Skromanis S, Cooling N, Rodgers B, Purton T, Fan F, Bridgman H, Harris K, Presser J, Mond J (2018). Health and well-being of international university students, and comparison with domestic students, in Tasmania, Australia. Int J Environ Res Public Health.

[5] Stallman H (2010). Psychological distress in university students: a comparison with general population data. Australian Psychologist.

[6] Clough BA, Nazareth SM, Day JJ, Casey LM (2018). A comparison of mental health literacy, attitudes, and help-seeking intentions among domestic and international tertiary students. British J Guidance Counselling.

[7] Lu SH, Dear BF, Johnston L, Wootton BM, Titov N (2013). An internet survey of emotional health, treatment seeking and barriers to accessing mental health treatment among Chinese-speaking international students in Australia. Counselling Psychol Q.

[8] Raunic A, Xenos S (2008). University counselling service utilisation by local and international students and user characteristics: a review. Int J Adv Counselling.

[9] Mellor D, Carne L, Shen Y, McCabe M, Wang L (2012). Stigma toward mental illness: a cross-cultural comparison of Taiwanese, Chinese immigrants to Australia and Anglo-Australians. J Cross Cultural Psychol.

[10] Ma S, Zhu Y, Bresnahan M (2022). Chinese international students' face concerns, self-stigma, linguistic factors, and help-seeking intentions for mental health. Health Commun.

[11] Newton DC, Tomyn AJ, LaMontagne AD (2021). Exploring the challenges and opportunities for improving the health and wellbeing of international students: perspectives of international students. J Australian New Zealand Student Services Assoc.

[12] (2020). International students and their mental health and physical safety. Orygen.

[13] Lian Z, Wallace BC, Fullilove RE (2020). Mental health help-seeking intentions among Chinese international students in the U.S. higher education system: the role of coping self-efficacy, social support, and stigma for seeking psychological help. Asian Am J Psychol.

[14] Gulliver A, Griffiths KM, Christensen H, Brewer JL (2012). A systematic review of help-seeking interventions for depression, anxiety and general psychological distress. BMC Psychiatry.

[15] Xu Z, Huang F, Kösters M, Staiger T, Becker T, Thornicroft G, Rüsch N (2018). Effectiveness of interventions to promote help-seeking for mental health problems: systematic review and meta-analysis. Psychol Med.

[16] Evans-Lacko S, Hahn J, Peter L, Schomerus G (2022). The impact of digital interventions on help-seeking behaviour for mental health problems: a systematic literature review. Curr Opin Psychiatry.

[17] Clough BA, Nazareth SM, Casey LM (2019). Making the grade: a pilot investigation of an e-intervention to increase mental health literacy and help-seeking intentions among international university students. Brit J Guidance Counselling.

[18] Ebert DD, Franke M, Kählke F, Küchler A-M, Bruffaerts R, Mortier P, Karyotaki E, Alonso J, Cuijpers P, Berking M, Auerbach RP, Kessler RC, Baumeister H, WHO World Mental Health - International College Student collaborators (2019). Increasing intentions to use mental health services among university students. Results of a pilot randomized controlled trial within the World Health Organization's World Mental Health International College Student Initiative. Int J Methods Psychiatr Res.

[19] Han J, Batterham P, Calear A, Wu Y, Xue J, van Spijker BA (2018). Development and pilot evaluation of an online psychoeducational program for suicide prevention among university students: a randomised controlled trial. Internet Interv.

[20] King CA, Eisenberg D, Zheng K, Czyz E, Kramer A, Horwitz A, Chermack S (2015). Online suicide risk screening and intervention with college students: a pilot randomized controlled trial. J Consult Clin Psychol.

[21] Taylor-Rodgers E, Batterham P (2014). Evaluation of an online psychoeducation intervention to promote mental health help seeking attitudes and intentions among young adults: randomised controlled trial. J Affect Disord.

[22] Tuliao A, Mullet N, Hawkins L, Holyoak D, Weerts M, Gudenrath T (2019). Examining the role of a brief online alcohol use risk feedback on accessing information about available treatment resources for alcohol issues. Addict Behav.

[23] Harper Shehadeh M, Heim E, Chowdhary N, Maercker A, Albanese E (2016). Cultural adaptation of minimally guided interventions for common mental disorders: a systematic review and meta-analysis. JMIR Ment Health.

[24] Rickwood D, Deane FP, Wilson CJ, Ciarrochi J (2005). Young people's help-seeking for mental health problems. Aust e JAdvancement Mental Health.

[25] Bakker D, Kazantzis N, Rickwood D, Rickard N (2016). Mental health smartphone apps: review and evidence-based recommendations for future developments. JMIR Ment Health.

[26] Peters D, Calvo RA, Ryan RM (2018). Designing for motivation, engagement and wellbeing in digital experience. Front Psychol.

[27] Kessler R C, Andrews G, Colpe L J, Hiripi E, Mroczek D K, Normand S L, Walters E E, Zaslavsky A M (2002). Short screening scales to monitor population prevalences and trends in non-specific psychological distress. Psychol Med.

[28] Andrews G, Slade T (2001). Interpreting scores on the Kessler Psychological Distress Scale (K10). Aust N Z J Public Health.

[29] Zhou C, Chu J, Wang T (2008). Reliability and validity of 10-item Kessler scale (K10) Chinese version in evaluation of mental health status of Chinese population. Chinese J Clin Psychol.

[30] (2012). 4817.0.55.001 - information paper: use of the Kessler psychological distress scale in ABS health surveys, Australia, 2007-08. Australian Bureau of Statistics.

[31] Bastien C, Vallières A, Morin C (2001). Validation of the Insomnia Severity Index as an outcome measure for insomnia research. Sleep Med.

[32] Chung K, Kan K, Yeung W (2011). Assessing insomnia in adolescents: comparison of Insomnia Severity Index, Athens Insomnia Scale and Sleep Quality Index. Sleep Med.

[33] Cohen S, Kamarck T, Mermelstein R (1983). A global measure of Perceived Stress. J Health Social Behav.

[34] Lu W, Bian Q, Wang W, Wu X, Wang Z, Zhao M (2017). Chinese version of the Perceived Stress Scale-10: a psychometric study in Chinese university students. PLoS One.

[35] Bush K, Kivlahan D R, McDonell M B, Fihn S D, Bradley K A (1998). The AUDIT alcohol consumption questions (AUDIT-C): an effective brief screening test for problem drinking. Arch Intern Med.

[36] Campbell CE, Maisto SA (2018). Validity of the AUDIT-C screen for at-risk drinking among students utilizing university primary care. J Am Coll Health.

[37] Li Q, Babor TF, Hao W, Chen X (2011). The Chinese translations of Alcohol Use Disorders Identification Test (AUDIT) in China: a systematic review. Alcohol Alcohol.

[38] Heatherton T F, Kozlowski L T, Frecker R C, Fagerström K O (1991). The Fagerström Test for Nicotine Dependence: a revision of the Fagerström Tolerance Questionnaire. Br J Addict.

[39] Huang C, Lin H, Wang H (2006). The psychometric properties of the Chinese version of the Fagerstrom Test for Nicotine Dependence. Addict Behav.

[40] Zimet GD, Dahlem NW, Zimet SG, Farley GK (1988). The multidimensional scale of perceived social support. J Personality Assessment.

[41] Zimet G, Powell S, Farley G, Werkman S, Berkoff K (1990). Psychometric characteristics of the Multidimensional Scale of Perceived Social Support. J Pers Assess.

[42] Guan NC, Seng LH, Hway Ann AY, Hui KO (2015). Factorial validity and reliability of the Malaysian simplified Chinese version of Multidimensional Scale of Perceived Social Support (MSPSS-SCV) among a group of university students. Asia Pac J Public Health.

[43] Proudfoot J, Clarke J, Birch M, Whitton AE, Parker G, Manicavasagar V, Harrison V, Christensen H, Hadzi-Pavlovic D (2013). Impact of a mobile phone and web program on symptom and functional outcomes for people with mild-to-moderate depression, anxiety and stress: a randomised controlled trial. BMC Psychiatry.

[44] Mahoney A, Elders A, Li I, David C, Haskelberg H, Guiney H, Millard M (2021). A tale of two countries: increased uptake of digital mental health services during the COVID-19 pandemic in Australia and New Zealand. Internet Interv.

[45] Fischer E, Farina A (1995). Attitudes toward seeking professional psychological help: a shortened form and considerations for research. J Coll Stud Dev.

[46] Elhai J, Schweinle W, Anderson S (2008). Reliability and validity of the attitudes toward seeking professional psychological help scale-short form. Psychiatry Res.

[47] Fang K, Pieterse AL, Friedlander M, Cao J (2011). Assessing the psychometric properties of the attitudes toward seeking professional psychological help scale-short form in Mainland China. Int J Adv Counselling.

[48] Wilson C, Deane F, Ciarrochi J, Rickwood D (2005). Measuring help seeking intentions: properties of the general help seeking questionnaire. Canadian J Counsel.

[49] Han J, Batterham P, Calear A, Ma J (2018). Seeking professional help for suicidal ideation: a comparison between Chinese and Australian university students. Psychiatry Res.

[50] Parks AC, Williams AL, Tugade MM, Hokes KE, Honomichl RD, Zilca RD (2018). Testing a scalable web and smartphone based intervention to improve depression, anxiety, and resilience: a randomized controlled trial. Int J Wellbeing.

[51] Peek J, Hay K, Hughes P, Kostellar A, Kumar S, Bhikoo Z, Serginson J, Marshall HM (2021). Feasibility and acceptability of a smoking cessation smartphone app (My QuitBuddy) in older persons: pilot randomized controlled trial. JMIR Form Res.

[52] Fleming T, Bavin L, Lucassen M, Stasiak K, Hopkins S, Merry S (2018). Beyond the trial: systematic review of real-world uptake and engagement with digital self-help interventions for depression, low mood, or anxiety. J Med Internet Res.

[53] Lattie EG, Adkins EC, Winquist N, Stiles-Shields C, Wafford QE, Graham AK (2019). Digital mental health interventions for depression, anxiety, and enhancement of psychological well-being among college students: systematic review. J Med Internet Res.

[54] Bedford L, Dietch J, Taylor D, Boals A, Zayfert C (2018). Computer-guided problem-solving treatment for depression, PTSD, and insomnia symptoms in student veterans: a pilot randomized controlled trial. Behav Ther.

[55] Haas A, Koestner B, Rosenberg J, Moore D, Garlow SJ, Sedway J, Nicholas L, Hendin H, Mann JJ, Nemeroff CB (2008). An interactive web-based method of outreach to college students at risk for suicide. J Am College Health.

[56] Horgan A, McCarthy G, Sweeney J (2013). An evaluation of an online peer support forum for university students with depressive symptoms. Arch Psychiatr Nurs.

[57] Lee RA, Jung ME (2018). Evaluation of an mHealth app (DeStressify) on university students' mental health: pilot trial. JMIR Ment Health.

[58] Pascoe M, Dash S, Klepac Pogrmilovic B, Patten R, Parker A (2022). The engagement of tertiary students with an online mental health intervention during the coronavirus disease 2019 pandemic: a feasibility study. Digit Health.

[59] Wasil AR, Taylor ME, Franzen RE, Steinberg JS, DeRubeis RJ (2021). Promoting graduate student mental health during COVID-19: acceptability, feasibility, and perceived utility of an online single-session intervention. Front Psychol.

[60] Melnyk B, Amaya M, Szalacha L, Hoying J, Taylor T, Bowersox K (2015). Feasibility, acceptability, and preliminary effects of the COPE online cognitive-behavioral skill-building program on mental health outcomes and academic performance in freshmen college students: a randomized controlled pilot study. J Child Adolesc Psychiatr Nurs.

[61] Kaczmarek L, Kashdan T, Kleiman E, Baczkowski B, Enko J, Siebers A, Szäefer A, Król M, Baran B (2013). Who self-initiates gratitude interventions in daily life? An examination of intentions, curiosity, depressive symptoms, and life satisfaction. Personality Individual Differences.

[62] Eustis E, Hayes-Skelton S, Orsillo S, Roemer L (2018). Surviving and thriving during stress: a randomized clinical trial comparing a brief web-based therapist-assisted acceptance-based behavioral intervention versus waitlist control for college students. Behav Ther.

[63] Lintvedt OK, Griffiths KM, Sørensen K, Østvik AR, Wang CE, Eisemann M, Waterloo K (2013). Evaluating the effectiveness and efficacy of unguided internet-based self-help intervention for the prevention of depression: a randomized controlled trial. Clin Psychol Psychother.

[64] Hintz S, Frazier PA, Meredith L (2015). Evaluating an online stress management intervention for college students. J Couns Psychol.

[65] Levin ME, Pistorello J, Seeley JR, Hayes SC (2014). Feasibility of a prototype web-based acceptance and commitment therapy prevention program for college students. J Am Coll Health.

[66] Choi I, Sharpe L, Li S, Hunt C (2015). Acceptability of psychological treatment to Chinese- and Caucasian-Australians: internet treatment reduces barriers but face-to-face care is preferred. Soc Psychiatry Psychiatr Epidemiol.

